# Supplementary material for: Genome-Wide CRISPR Screens Identify Genome Stability as Regulator of the Cellular Sensitivity to Environmentally Relevant Bisphenol A Exposure
Source: Biomolecules. 2026 Sep 9;16(9):1303. doi: 10.3390/biom16091303 (PMC13604100; doi:10.3390/biom16091303)
Supplement: Supplementary file 1 [file biomolecules-16-01303-s001.zip › biomolecules-4503116-supplementary.pdf]

Supplementary Material

Legends to Supplementary Tables

Table S1. MAGeCK analyses of the CRISPR screens identifying genes whose loss confers increased sensitivity to BPA in HeLa cells.

Table S2. MAGeCK analyses of the CRISPR screens identifying genes whose loss confers increased sensitivity to BPA in RPE1 cells.

Table S3. List of common hits with MAGeCK score lower than 0.01 from the CRISPR knockout screens for BPA sensitivity in HeLa and RPE1 cells.

Table S4. The source data underlying each of the main and supplementary figure panels, including the values plotted in graphs and the exact p-values.

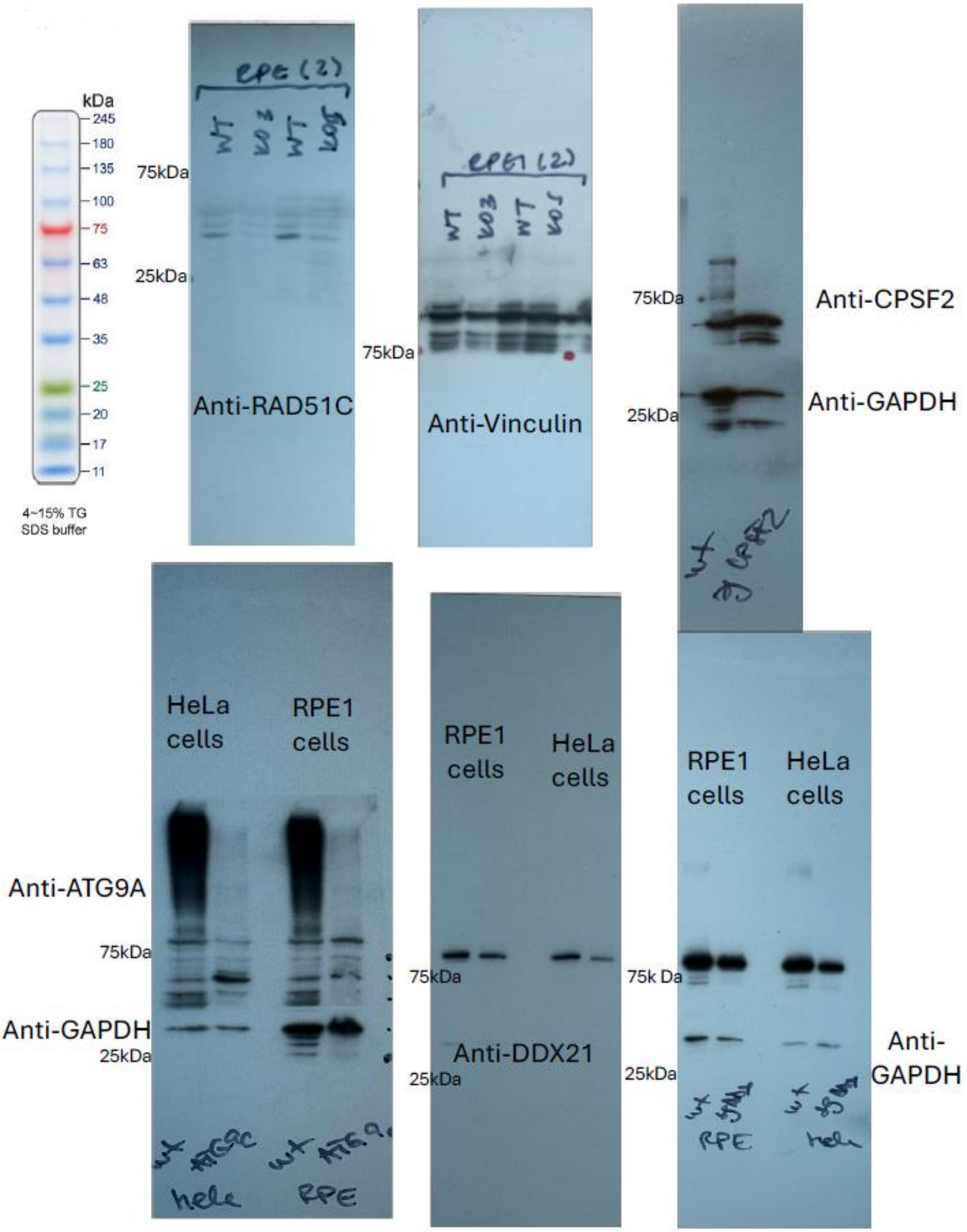

Figure S1. Uncropped blots of the western blots shown in Figure 4A.

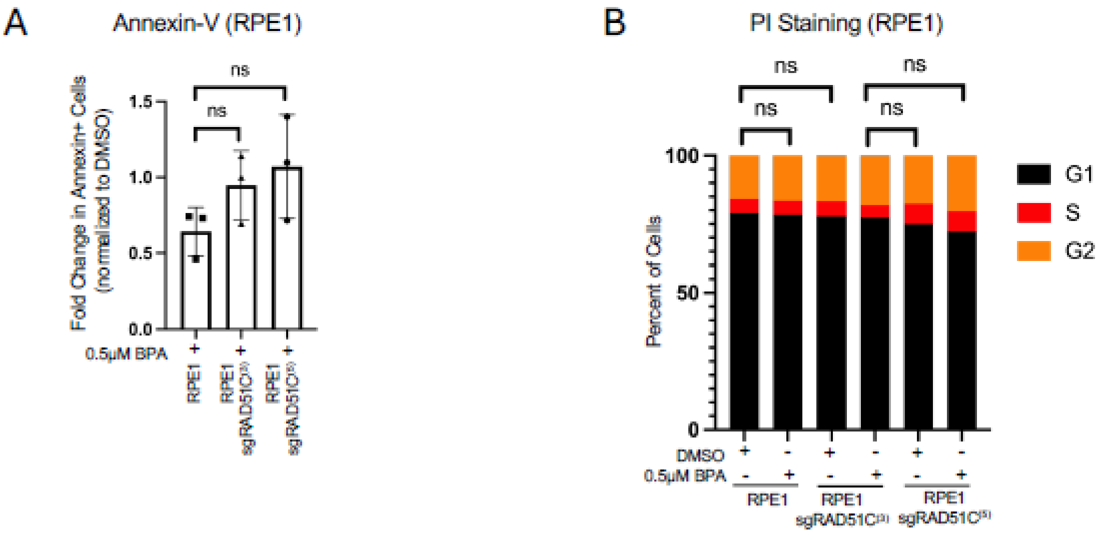

Figure S2. Impact of RAD51C depletion on the cell cycle upon BPA treatment. (A) Annexin-V staining showing the impact of sgRAD51 on apoptosis upon BPA treatment in RPE1 cells. Three independent experiments are shown (t-test two-tailed, unpaired). (B) PI staining showing the impact of sgRAD51 on apoptosis upon BPA treatment in RPE1 cells. Three independent experiments are shown (One-way ANOVA, Tukey's Multiple Comparisons test).

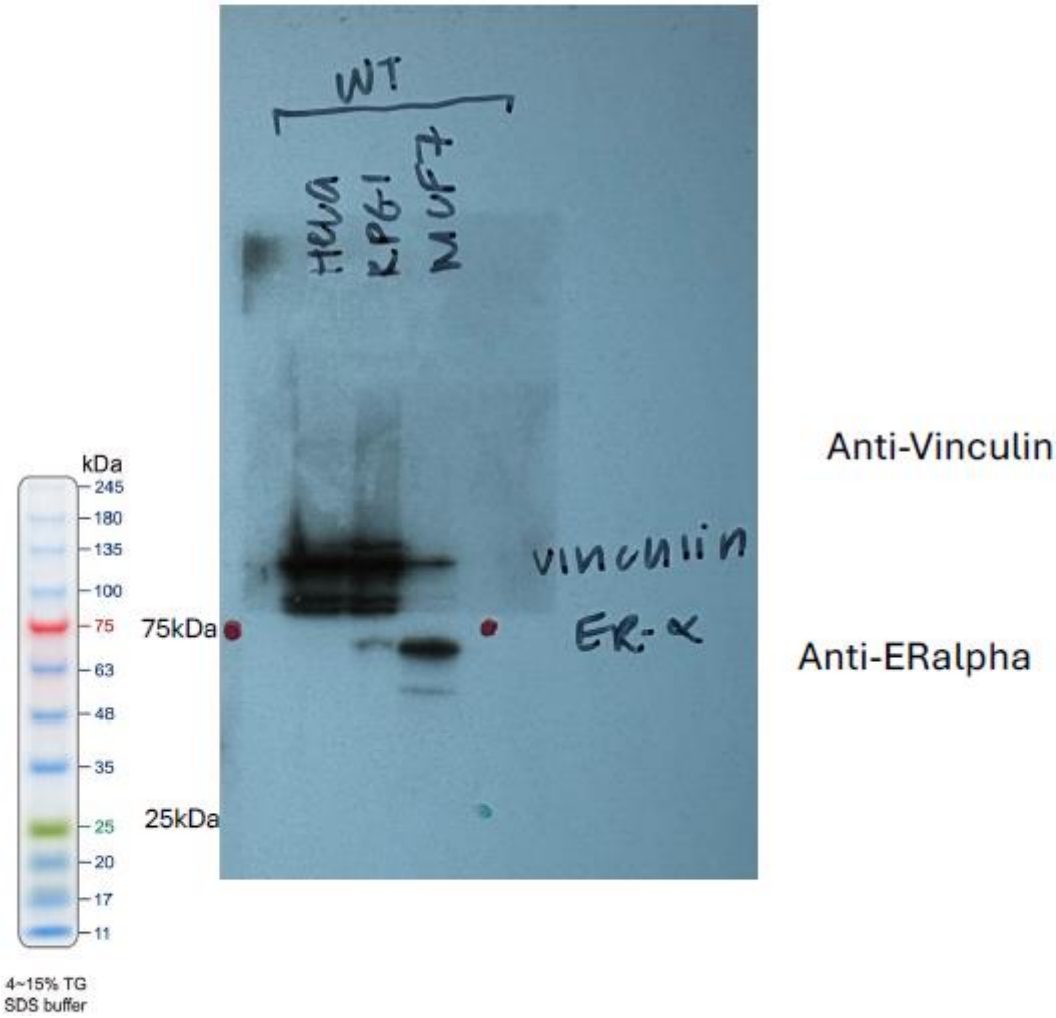

Figure S3. Uncropped blots of the western blots shown in Figure 6C.
